# Physical activity in metabolic syndrome

**DOI:** 10.3389/fphys.2024.1365761

**Published:** 2024-02-19

**Authors:** Tomasz Chomiuk, Natalia Niezgoda, Artur Mamcarz, Daniel Śliż

**Affiliations:** 3rd Department of Internal Medicine and Cardiology, Medical University of Warsaw, Warsaw, Poland

**Keywords:** obesity, physical activity, metabolic syndrome, insulin resistance, hypertension

## Abstract

Obesity has become one of the global epidemics, contributing to the burden of disease in society, increasing the risk of diabetes, cardiovascular and liver diseases. Inadequate energy balance resulting from excessive energy intake and insufficient physical activity (PA) is one of the main factors contributing to the incidence of obesity and the development of metabolic syndrome (MetS). Treatment options for obesity include lifestyle modifications, pharmacotherapy and bariatric surgery, with the latter being the most effective treatment. Lifestyle interventions involving increased PA and reduced caloric intake improve metabolic outcomes. Early implementation of exercise leads to improved physical fitness, better glycemic control and lipid profile. Undertaking systematic PA is associated with better quality of life, improves insulin sensitivity, causes additional weight loss, reduces its adverse effects on bone mass and results in better body composition. In this narrative review we summarized the current state of knowledge on the impact of PA on the components of MetS and the latest recommendations for PA in patients with MetS.

## 1 Introduction

Metabolic Syndrome (MetS) is one of main public health problems of recent years. The presence of MetS significantly increases the risk of diabetes and cardiovascular disease (Rochlani et al., 2017; Hsu et al., 2021). The MetS is not a single disease, but a set of risk factors for cardiovascular disease, the criteria for which have evolved over the years and have been defined differently by health organizations (Saklayen, 2018). The US National Cholesterol Education Programme Adult Treatment Panel III (NCEP ATP III) and International Diabetes Federation (IDF) definitions take the presence of obesity, dyslipidemia, elevated blood pressure and elevated fasting glucose levels as diagnostic criteria (Grundy et al., 2005; Alberti et al., 2006). According to the latest definition by Polish Society of Hypertension, which takes into account both other definitions and the latest management guidelines of individual components of MetS, the diagnostic criteria for MetS include the presence of obesity and two of the three following criteria: high blood pressure, impaired glucose metabolism, and elevated levels of low-density lipoprotein (LDL) and non HDL cholesterol (non-HDL). The main diagnostic criterion for MetS is obesity, which is diagnosed by a waist circumference greater than 88 cm in women and 102 cm in men or a body mass index (BMI) > 30 kg/m2 (Dobrowolski et al., 2022). Current diagnostic criteria for insulin resistance assume a fasting glucose level of 100–125 mg/dL, 140–199 mg/dL after 120 min in an oral glucose tolerance test, HbA1C 5.7%–6.4% according to the American Diabetes Association (ADA) (American Diabetes Association Professional Practice Committee, 2022). Another of the additional diagnostic criteria is an elevated non-HDL cholesterol level of >130 mg/dL, which measures the cholesterol content of all atherogenic lipoproteins, including LDL (Virani, 2011). A third additional diagnostic criterion is a normal systolic blood pressure greater than or equal to 135 mmHg and a diastolic blood pressure of 85 mmHg measured in the office or a systolic >130 mmHg and diastolic >80 mmHg measured at home. In addition, therapy with glucose-lowering drugs, cholesterol-lowering drugs or blood pressure also qualify as additional diagnostic criteria (Dobrowolski et al., 2022). The multisystem nature of MetS results from overlapping inflammation, oxidative stress, hemodynamic dysfunction and ischemia in patients. This results in an increased risk of cardiovascular disease, non-alcoholic fatty liver disease and other liver dysfunctions, chronic kidney disease, cancer and neurodegenerative disorders (Silveira Rossi et al., 2022).

The pathogenic mechanisms associated with MetS are complex and need to be fully elucidated. There is still debate as to whether the various components of the MetS represent separate pathologies or are manifestations of a common pathogenic mechanism. The large geographic variation in the prevalence of MetS emphasizes the importance of environmental and lifestyle factors, such as excess dietary calories and physical inactivity, as major contributors to the disease. Adipose tissue secretes cytokines that contribute to insulin resistance and endothelial dysfunction that cause the development of MetS (Van Alsten et al., 2020). Studies show that abdominal obesity is a key trigger for most of the pathways associated with MetS, emphasizing the importance of excess caloric intake as a major initiating factor (Matsuzawa et al., 2011). Of all the proposed mechanisms, insulin resistance, neurohormonal activation and chronic inflammation appear to be the main factors leading to the development of MetS and cardiovascular disease (Rochlani et al., 2017).

The number of people diagnosed with MetS is increasing, especially in developed countries. It is estimated that the prevalence of MetS can exceed as much as 30% of the population, depending on origin (Noubiap et al., 2022). Prevalence varies according to age, gender, race, ethnicity and diagnostic criteria. The MetS affects one-fifth or more of the US population and about one-quarter of the European population (Rochlani et al., 2017; Saklayen, 2018). Rapid economic growth and globalization among others are the reasons for the observed increase in the prevalence of MetS. Regardless of regional wealth, an unhealthy diet and insufficient levels of PA have become common worldwide (Noubiap et al., 2022).

Metabolic Syndrome is now being observed in the pediatric population. The most common cause of MetS in general population is hyperlipidemia, which affects 60% of polish population. Second, most common is obesity, which affects people of all ages and their occurrence is increasing since last 2 decades. The most important is that according to a systematic review of 85 studies involving children, the median prevalence of MetS in all populations was 3.3% (Friend et al., 2013).

Children with obesity with MetS are characterized by poor physical performance and poor eating habits. They often have glucose intolerance with insulin resistance. Genetic factors, gender and birth weight also influence the incidence of MetS in children—subjects with low birth weight were more likely to have MetS (Xiao et al., 2010; Jankowska et al., 2021). However, it is lifestyle, technology, inadequate PA, prolonged sitting, and a diet that includes fast food, snacks, sugary drinks and other products high in sugar and fat that have the greatest impact on the development of MetS (Jankowski et al., 2015; Ishaque, 2021).

The prevalence of MetS increases with age and gender differences have been observed (Hirode and Wong, 2020; Lind et al., 2021). The presence of MetS at a young age increases the relative risk of cardiovascular disease. This risk, in turn, decreases with the age of diagnosis of MetS (Huang et al., 2022).

Correct diagnosis and therapy of MetS is therefore highly relevant to preventive measures at the individual and population levels, in which the incidence of the disease is increasing in frequency and across all age groups. The purpose of this narrative review is to present the impact of PA on the components of MetS and to summarize recommendations for different types of PA in patients with MetS.

## 2 Physical activity in people with metabolic syndrome—physiologicalbasis

**FIGURE 1:**
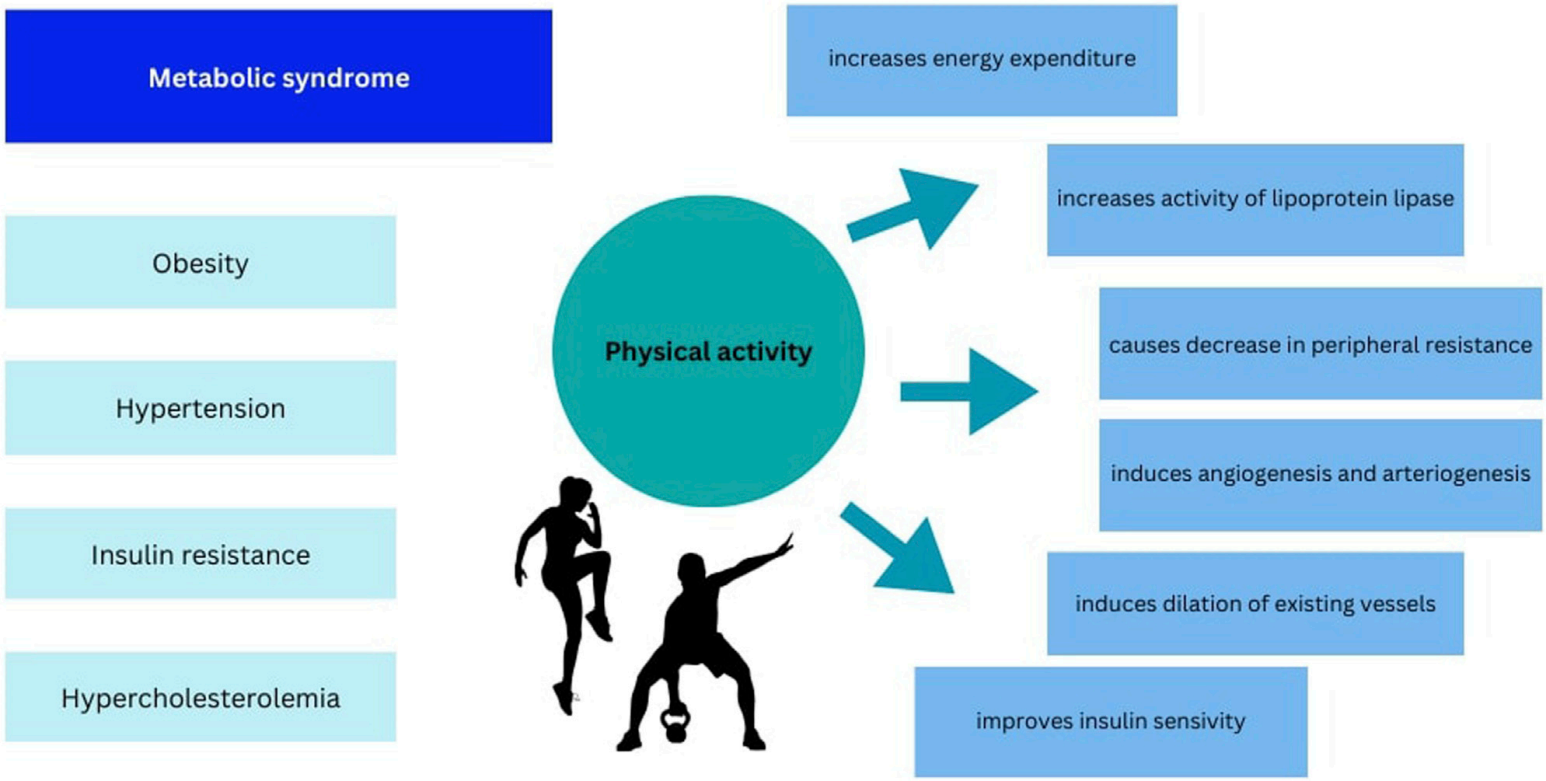
Summary of the effect of physical activity on components of metabolic syndrome.

Each clinical component of MetS can be modified by physical activity (PA) what is shown in Figure 1. PA is defined as any bodily movement produced by skeletal muscles that require energy expenditure. Exercise however is considered a subcategory of PA that is repetitive, planned, structured and purposeful (Dasso, 2019). Regular, moderate PA contributes to improving insulin sensitivity, lipid profile, blood pressure and body composition (Caro et al., 2013; Weiss et al., 2017; Shariful Islam et al., 2023). On the other hand low levels of physical fitness is one of the major risk factors for MetS and overall mortality (Hong et al., 2014; Blaha et al., 2016).

Oxidative stress and inflammation play an important role in the development of all the individual components of the MetS (Golbidi et al., 2012).

The anti-inflammatory effect of regular exercise is associated with a decrease in visceral fat mass and therefore a decrease in the release of pro-inflammatory adipokines (Gleeson et al., 2011). The anti-inflammatory effects of exercise may also be independent of changes in fat mass. PA has been shown to induce anti-inflammatory cytokines, such as IL-1 receptor antagonist (IL-1RA) and soluble IL-10, while reducing the production of the pro-inflammatory cytokines IL-1β and TNF-α (Allen et al., 2015).

The beneficial effects of exercise are also reflected in the release of myokines—cytokines, interleukins such as IL-6 and other peptides produced by muscle fibers. They participate in protection against inflammation-related diseases, including atherosclerosis. The anti-inflammatory effects of exercise training may also result from modulation of intracellular signaling pathways mediated by nitric oxide (NO) and oxygen free radicals. Increased production of NO and oxygen free radicals during training is important for inducing anti-inflammatory defense mechanisms (Nishii et al., 2023). Oxygen free radicals exhibit both beneficial and toxic effects. When their excess cannot be gradually recycled or when the body’s naturally occurring defenses are weak, the accumulation of free radicals causes a phenomenon called “oxidative stress” (Scheele et al., 2009; Simioni et al., 2018). Regular exercise has been shown to allow cells to better detoxify large amounts of reactive oxygen species both in adults and in older adults, who have antioxidant activity similar to young people with sedentary lifestyles and who can benefit from PA to protect themselves from oxidative damage and prevent age-related disorders (Simioni et al., 2018).

### 2.1 Physical activity and obesity

Decreased PA is strongly associated with increased risk of obesity (Nunn et al., 2010). Increasing energy expenditure with decreased intake can help reduce excess body fat and obesity. Scientific reports indicate that methods that can effectively reduce body fat include changing diet and modifying energy expenditure through exercise (Niemiro et al., 2023). Visceral fat is one of the causes of systemic inflammation, which leads to insulin resistance, type II diabetes and atherosclerosis (Ellulu et al., 2017). An inverse relationship is noted between PA, BMI, hip to waist ratio and waist circumference. Weight reduction through physical training results in less loss of muscle mass compared to body fat than weight loss through diet alone (Weiss et al., 2017; Shozi et al., 2022). It has been observed that lean body mass accounts for a large portion of insulin-stimulated glucose uptake, whence the assumption that greater lean body mass has a better effect on glucose homeostasis. Moreover, considering the relationship between lean body mass and resting energy expenditure, higher lean body mass has a protective effect against excessive fat accumulation through higher resting energy expenditure (Lagacé et al., 2022). In addition, reduction in fat mass promotes increased adiponectin levels and im-proved cytokine profiles, changes in which are associated with MetS and the development of insulin resistance. In a study by Venojärvi et al. (2022) a 2-year diet and exercise intervention reduced leptin levels and increased adiponectin levels in the subjects. They also observed improved glucose control without affecting GLUT-4 gene expression in skeletal muscle in the subjects. In addition, improved insulin sensitivity was associated with improved maximal oxygen uptake (VO2max) (Venojärvi et al., 2022).

### 2.2 Physical activity and hypercholesterolemia

Exercise improves the ability of skeletal muscle to utilize fats as opposed to glycogen, resulting in lower plasma lipid levels (Mann et al., 2014). A known mechanism that improves the lipid profile is the increased activity of lipoprotein lipase (LPL) under the influence of PA, which is responsible for the hydrolysis of chylomicrons and VLDL (Crichton and Alkerwi, 2015). In a study by Caro et al. (2013) significant differences in lipid parameters (triglicerydes, HDL-C, non-HDL-C, apoliprotein B) were observed between those with sedentary lifestyles and those who exercised regularly (at an intensity of 7.5–15 METs per week). Similar trends were observed in the Crichton & Alkrewi study. Increased screen time negatively affected lipid pro-file, particularly HDL levels regardless of age, gender, education, occupation type, income, PA, dietary factors and smoking (Crichton and Alkerwi, 2015).

### 2.3 Physical activity and hypertension

Reducing systolic blood pressure by 10 mmHg and diastolic blood pressure by 5 mmHg can reduce cardiovascular incidents by a quarter, stroke by a third and mortality from any cause by 13% (Ettehad et al., 2016). Mechanisms through which physical training and weight reduction affect blood pressure include structural and functional changes in the vascular system, modulation of the renin-angiotensin system, decreased stimulation of the sympathetic nervous system and increased insulin sensitivity (Golbidi et al., 2012). Physical training has significant effects on the morphology of various blood vessels. It causes vascular remodeling and increases the cross-sectional area and diameter of veins and arteries. This results in a decrease in peripheral resistance (Stebbings et al., 2013). In addition to structural changes, there are functional changes that lead to improved blood flow. Exercise induces angiogenesis, or the formation of new blood vessels at the level of capillary resistance arterioles, and arteriogenesis, or the dilation of existing vessels (Königstein et al., 2023). Exercise, moreover, reduces the vascular response to endothelin-1, which is a vasoconstrictor in people with established hypertension. Physical training, by increasing nitric oxide production and acetyl-choline release, increases endothelium-dependent vasodilation (Rêgo et al., 2019; Shariful Islam et al., 2023). Additionally, in people with hypertension PA reduces sympathetic nerve activity (Shariful Islam et al., 2023). The sympathetic nervous system is activated during exercise, but regular training can reduce the activity of the sympathetic nervous system. Slow breathing after exercise stimulates parasympathetic nervous system which regulates autonomic balance (Daniela et al., 2022).

### 2.4 Physical activity and insulin resistance

In people with diabetes caused by insulin resistance, beneficial changes in glucose tolerance can be made through regular exercise. Abdominal obesity contributes to insulin resistance and regular exercise helps to reduce body fat, thereby increasing cellular sensitivity to insulin (Hong et al., 2014; Shih and Kwok, 2018). The weight loss influenced by PA improves the oxidative capacity of mitochondria and significantly inhibits gluconeogenesis by affecting endogenous glucose production. The effectiveness of sustained improvements in glucose metabolism may be influenced by the intensity of exercise, as it is associated with changes in body composition—in fat mass, visceral and subcutaneous adipose tissue and percentage of fat mass, which may translate into improvements in glucose tolerance (Keshel and Coker, 2015). Scientific reports suggest that a sedentary lifestyle affects changes in muscle glucose transporter protein (GLUT) which affects carbohydrate metabolism. Moreover, skeletal muscle denervation causes a rapid decrease in both muscle GLUT-4 content and insulin-stimulated glucose uptake (Strasser, 2013). In conclusion, insufficient levels of PA may contribute to the development of insulin resistance by decreasing the efficiency of pancreatic β-cells through various pathways—including mitochondrial dysfunction, oxidative stress and inflammation, and apoptosis (Hudish et al., 2019; Yaribeygi et al., 2021).

## 3 Recommended forms of physical activity in people with metabolic syndrome

PA and an active lifestyle have a preventive effect in the context of oxidative stress, but also in primary and secondary protection against cardiovascular disease, type II diabetes, MetS and neurodegenerative diseases, including Alzheimer’s disease (Simioni et al., 2018).

### 3.1 Aerobic training

Aerobic training (AT) is the most effective type of exercise in health problems as-sociated with MetS. A study by Bateman et al. (2011) found that AT improved MetS parameters to a greater extent than resistance training (RT). However, the greatest improvement was seen with a combination of resistance and aerobic exercise (Bateman et al., 2011).

AT that causes energy expenditure is an important tool in reducing body weight and body fat, including visceral fat. Best results are obtained when combined with a balanced diet. Intervention with AT alone results in a small decrease in body weight (0–2 kg) and its effectiveness is possible only with high training volumes (Donnelly et al., 2009; Swift et al., 2014).

AT is one of the main lifestyle interventions introduced in hypertensive patients. It has been observed in a meta-analysis that AT lowers systolic blood pressure by 8–12 mmHg and diastolic blood pressure by 5–6 mmHg in hypertensive adults (de Barcelos et al., 2022). This is associated with, among other things, a reduction in arterial stiffness, effects on auto-nomic function, as well as improved endothelial function and reduced inflammation of the vessel wall (Lopes et al., 2018). AT is also an effective intervention in achieving control of systolic and diastolic blood pressure in patients with uncontrolled hypertension using two antihypertensive drugs. This was demonstrated in a study by Maruf et al. (2016) in which subjects underwent training program–12 weeks of aerobic dance exercise at 50%–70% intensity 3 times a week. The researchers concluded that AT may be useful in treating people with hypertension without the need to prescribe a third blood pres-sure-lowering drug (Maruf et al., 2016).

Another systematic review and meta-analysis indicates that PA of various types at moderate intensity performed during leisure time has a significant effect on lowering blood pressure (both systolic and diastolic) compared to a non-intervention control group (Shariful Islam et al., 2023).

AT has beneficial systemic effects. The study by Monda et al. (2020) checked several blood parameters [aspartate aminotransferase (AST), alanine aminotransferase (ALT), gamma-glutamyl transpeptidase (GGT), total cholesterol (TC), HDL, LDL and TG] after 6 months of regular AT with no changes in diet. All blood parameters studied improved from baseline levels. An association between increased plasma orexin A levels and PA was also described. This neuropeptide plays an important role in key states: sleep-wakefulness, eating behavior, mood or energy homeostasis. It has been shown to be involved in adaptations to exercise (Monda et al., 2020).

The findings suggest that the timing, volume and intensity of exercise affect changes in blood lipid levels. HDL-C is most sensitive to exercise. To lower LDL-C and TG levels more, it is necessary to increase the intensity of AT. Although high-intensity AT has a very significant effect on improving the lipid profile (especially LDL-C and TG), moderate or lower intensity exercise is sufficient for most people. Many people with MetS should not be subjected to high-intensity exercise (Wang and Xu, 2017).

Since muscle contractions occurring during exercise increase glucose uptake in skeletal muscle, it is recommended for patients with type 2 diabetes. Significant differences were observed in fasting blood glucose, plasma insulin levels and insulin resistance in patients with type II diabetes after an 8-week AT intervention at an intensity of 60% compared to a control group (Motahari-Tabari et al., 2014). According to recommendations for people with type II diabetes, for the greatest health benefits, moderate to high intensity exercise should be at least 150 min per week. AT can be performed continuously or as high-intensity interval training (HIIT), which is characterized by short intense bursts interspersed with recovery periods. Similar metabolic and cardioprotective benefits can be obtained by younger or more physically fit patients performing HIIT vigorously for 75 min/week. However, this type of training is recommended for clinically stable patients who are already performing vigorous PA (Cannata et al., 2020).

### 3.2 Resistance training

Resistance training (RT) increases strength, muscle mass and lean body mass more than AT. However, because of the increase in muscle mass, it does not cause weight loss without caloric restriction. But, even without caloric restriction, it has a beneficial effect on body composition because it reduces fat mass, including abdominal fat, and increases basal metabolism. At rest, skeletal muscle consumes 54.4 kJ/kg (13.0 kcal/kg) per day, more than adipose tissue-18.8 kJ/kg (4.5 kcal/kg) (Heymsfield et al., 2002; Sundell, 2011). This is particularly important for people losing weight, as resting metabolism is reduced after weight loss in healthy normal weight and overweight individuals. This reduction occurs due to the loss of mass of energy-expendable tissues and metabolic adaptations. Consequently, the loss of energy expendable tissues—mainly skeletal muscle and adipose tissue—contributes to a reduction in resting metabolism (Martin et al., 2022).

RT has also been shown to increase insulin sensitivity, improve glucose tolerance and lower blood pressure values (Irvine and Taylor, 2009; Sundell, 2011). A meta-analysis by Jiahao et al. (2021) showed that resistance training improves insulin sensitivity in the elderly. High-intensity, long-term exercise had a better effect on improving indices related to insulin resistance in healthy individuals, while shorter, moderate-intensity activities are preferred in people with type II diabetes (Jiahao et al., 2021). A meta-analysis by de Sousa et al. (2017) showed that a RT intervention lowered both systolic and diastolic blood pressure in prehypertensive and hypertensive subjects. The hypotensive effect of RT may be due to reduced peripheral resistance and improved endothelial function (de Sousa et al., 2017). Correia et al. (2023) observed that the strongest effect of RT on lowering blood pressure is exerted by exercise with moderate to heavy loads >60% of one repetition maximum (1RM), frequency at least twice a week and performed for at least 8 weeks (Correia et al., 2023). In a study by Ihalainen et al. (2019) it was shown that in elderly patients (65–75 years old), RT performed more often than 2 times a week has a beneficial effect on lipid profile and body composition. In addition, patients with higher baseline systolic blood pressure, triglycerides and hs-CRP levels benefited the most from RT, regardless of frequency of exercise (Ihalainen et al., 2019). Low/moderate in-tensity RT (≤75% of 1RM) induces more beneficial changes in lipid profile than high-intensity RT (Lira et al., 2010).

### 3.3 Recommendations for exercise in people with metabolic syndrome

Moderate intensity AT is particularly recommended to reduce body weight, visceral fat and improve blood pressure. To maintain lean body mass during weight loss, it is recommended to include moderate to high intensity RT in the exercise program. To improve insulin sensitivity and increase cardiorespiratory fitness, any type of training is appropriate including HIIT implemented under supervision after a thorough cardiovascular risk assessment (Oppert et al., 2021).

Despite the benefits of high intensity training for most people, especially patients with cardiovascular disease, high intensity exercise is not recommended. To improve health parameters, moderate-intensity exercise is sufficient. In untrained individuals, even light exercise produces beneficial effects, and the metabolic benefits of PA are observed even in the absence of significant weight loss (Mann et al., 2014; Joseph et al., 2019). In addition, it is recommended to reduce the amount of time spent sitting. Katzmarzyk et al. (2009) observed an association between sitting time and all-cause and cardiovascular mortality, regardless of the level of leisure-time PA.

Attention is being paid to the role of non-exercise activity thermogenesis (NEAT) that represents spontaneous PA in the prevention of components of the MetS, most notably obesity. NEAT is crucial for regulating energy expenditure. It is a highly variable component of daily total energy expenditure and low NEAT levels are associated with the incidence of obesity. NEAT levels are highly dependent on individual and environmental factors including work and leisure time. It also includes going to work/school or fulfilling household chores such as cleaning, cooking or gardening (Villablanca et al., 2015; Chung et al., 2018).

The appropriate amount of PA for adults recommended by the WHO is at least 150 min per week of moderate-intensity aerobic exercise or 75 min per week of high-intensity aerobic exercise, or a combination of both. In addition, it is recommended to implement muscle strengthening training (resistance or weight training) of moderate to high intensity for at least 2 days a week. Recommendations also include limiting time in a sedentary position in favor of even low-intensity activity. More health benefits can be gained by being active for at least 300 min a week (Organization, 2022). Re-duced PA aggravated by technological advances and an increasing sedentary lifestyle promotes the development of MetS. Summary recommendations for PA in the prevention of cardiovascular diseases based on European Society of Cardiology (ESC) and American College of Cardiology/American Heart Association (ACC/AHA) are provided in Table 1 (Arnett et al., 2019; Visseren et al., 2021).

**TABLE 1:** Recommendations for physical activity in the prevention of cardiovascular diseases.

Type of PA	Frequency	Intensity	Activities
Aerobic training	150–300 min/week moderate intensity or	3–5,9 MET	Vacuuming, gardening, brisk walking, ballroom dancing, water aerobics, tennis (doubles), cycling 15 km/h
	75–150 vigourous intensity	≥6 MET	Running, aerobic dancing, tennis (singles), jumping rope, heavy yardwork (digging), cycling >15 km/h
Resistance training	At least 2 times/week	60%–80% RM	Weight—bearing exercises, bars, discs, dumbbells, kattlebells, resistance machines
	1–3 sets		
	8–12 reps		
	8–10 different exercises		

HRR, heart-rate reserve; min, minutes; reps, repetitions; RM, repetition maximum.

In Bankoski et al. (2011) study, people with MetS compared to healthy individuals spent more time sitting (67.3% vs. 62.2%). More time spent in sedentary position and fewer sitting breaks were associated with a significantly higher likelihood of MetS after adjusting for age, gender, education, ethnicity, alcohol consumption, smoking, BMI, prevalence of diabetes and heart disease, and PA level (Bankoski et al., 2011).

Studies show that meeting or exceeding PA recommendations is inversely related to the risk of MetS and improves parameters in people with already established MetS or its components (Mann et al., 2014; Wu et al., 2016; Joseph et al., 2019).

## 4 Conclusion

MetS is a condition affecting more and more people worldwide. All components of metabolic syndrome can be modified through lifestyle changes, primarily through changes in diet and physical activity. With various forms of PA, a wide selection of intensities and types, it is available to patients of all ages and physical conditions. Each clinical component of the metabolic syndrome can be modified with physical activity, which is a low-cost and therefore easily accessible way to prevent and treat the metabolic syndrome.

## Abbreviations

PA, physical activity; MetS, metabolic syndrome; AT, aerobic training; RT, resistance training; HIIT, high-intensity interval training; NEAT, non-exercise activity thermogenesis.
